# Music as an Element of Tourism Innovation: Types of Nightlife Premises in Ibiza (Spain)

**DOI:** 10.3389/fpsyg.2022.890847

**Published:** 2022-06-17

**Authors:** José Ramón-Cardona, María Dolores Sánchez-Fernández, Amador Durán-Sánchez, José Álvarez-García

**Affiliations:** ^1^Escuela Universitaria de Turismo del Consell Insular de Ibiza, Universidad de las Islas Baleares, Palma, Spain; ^2^Departamento de Empresa, Universidad de la Coruña, A Coruña, Spain; ^3^Departamento de Economía Financiera y Contabilidad, Facultad de Empresa Finanzas y Turismo, Instituto Universitario de Investigación para el Desarrollo Territorial Sostenible (INTERRA), Universidad de Extremadura, Avenida de la Universidad, Cáceres, Spain

**Keywords:** Ibiza, nightlife, offer, types, establishment, entrepreneur

## Abstract

The island of Ibiza is a western Mediterranean destination known internationally for its nightlife. The aim of this paper is to make a proposal to classify the different types of premises in the Ibiza nightlife offer. This involves making a first definition that allows to delimit which businesses are parts of the sector. The methodology used is based on the case study and specifically, on the review of the promotional actions and activities carried out, completed with the visit to the premises. The classification has been made based on the offer marketed and not only on the legal forms used, as innovation goes ahead of the existing legal classifications. Although it is a particular application, due to the international importance of Ibiza, it is a good starting point to classify the nightlife offer of many other tourist destinations. The resulting typology divides the sector into two large groups: nightclubs and other premises. While nightclubs have musical parties as their main activity declared, the other premises have accommodation or catering as their main activity, with music being an element of differentiation. Nightclubs are divided into several subgroups, depending on their size and relevance. The other premises are subdivided into Beach Clubs, Hotel Clubs, Party Boats, Lounge Clubs, Disco Pubs, among others.

## Introduction

Ibiza is an island in the Western Mediterranean Sea, with a surface of 572 km^2^ and more than 160,000 inhabitants (IBESTAT, 2022), characterized by its strong economic dependence on tourism. Every year it receives about three million tourists, mostly concentrated in the months of June–September. The main tourist source markets are the United Kingdom (26.6%), Spain (20.9%), Italy (13.2%), and Germany (9.6%), being the rest minority markets (AETIB, 2021). The island’s offer has been based on sun and beach tourism since the 1950s, but its image was linked to the concept of freedom which accompanied the countercultural movements that passed through the island in the years of the Tourism Boom, mainly beatniks and hippies (Ramón and Ramón, 2021). This idea of freedom and the traditions attributed to the countercultural collectives evolved into the different elements, which have made it possible to differentiate the island: Adlib fashion, craft markets, nightlife, etc. Therefore, the nightlife offer is an evolution of the idea of freedom linked to the island of the 1960s and 1970s (Ramón and Sánchez, 2022).

From the 1970s onwards, venues and entrepreneurs specializing in nightlife emerged, reaching high levels of international repercussion in the 1980s and re-emerging strongly in the late 1990s, after a major crisis and sector reforms in the first half of the 1990s (Ramón and Ramón, 2021). Nowadays, the nightlife is concentrated in several areas of the island (Sant Antoni, port of Ibiza, Platja d’en Bossa, and Sant Rafel) and it should be noted that the greatest media impact is due to a small group of large nightclubs of worldwide relevance (Amnesia, Pacha, Ushuaïa, Hï, Privilege, etc.).

It is an important sector for the local economy, but its greatest importance is for the image and differentiation of Ibiza as a tourist destination. Currently, beaches and nightclubs are the two most characteristic elements of the island’s image (Berrozpe et al., 2017), although in recent years other elements have been introduced, such as glamour and celebrities, which provide an expensive and luxurious destination component (Ramón and Sánchez, 2022).

There is still little academic literature on the management of the nightlife sector. The bibliography regarding nightclubs is more extensive as a context for the study of various social aspects, mainly social and health problems popularly attributed to nightlife. This paper aims to propose a classification of the current event venues on the island of Ibiza. The usefulness of making a classification proposal is to clarify which premises are direct competitors and which are complementary; which allows to reflect the existence of an offer beyond nightclubs and pubs. This complexity is due to the enormous number of innovative business initiatives that have emerged in the last four decades, which combine different services with musical events. It should be noted that the international success of Ibiza’s nightlife is due to the tradition of innovation and reinvestment of many nightlife entrepreneurs.

The proposed classification is useful for both entrepreneurs and researchers, focusing their studies and actions toward a specific type of offer. The premises under study have nightlife, specifically the celebration of parties or musical events as a characteristic element of their offer. In some cases, the events are the main activity (nightclubs) and in other cases, complementary and differentiating from their main activity, but in all cases, it implies having the basic equipment for holding musical events, mainly a mixing console and sound equipment for the main room. Those premises that hold events on a very sporadic basis, renting the necessary equipment in each case, and cases in which the events are not part of the regular offer and promotion are excluded from this classification.

Following a brief review of the literature, the history of nightlife in Ibiza and its current importance are discussed. It should be noted that for many years, only bars and nightclubs made up this offer, but at the end of the 1990s, beach clubs appeared and as of 2010, the other formats shown. The methodology used in this paper is discussed in section three. This is followed by the proposed classification and, finally, a number of conclusions.

### State of the Art on Nightlife

Most of the academic literature considers nightclubs to be a mere context or setting for studies not directly related to business management or marketing. Examples of this are various studies on the use and abuse of alcohol, drugs, and tobacco (Allen et al., 2020; Labhart et al., 2020; Palamar and Keyes, 2020; Jorgenrud et al., 2021), violence, assaults, and security (Carlini and Sánchez, 2018; Sánchez et al., 2019; Quigg et al., 2020; Fung et al., 2021), risky sexual relationships (Buttram et al., 2018; Carlini and Sánchez, 2018; Palamar et al., 2018), disasters and accidents (Crestani Calegaro et al., 2019; Meyer, 2020; Smith et al., 2020), such as the Pulse nightclub shooting (Gavulic and Gonzales, 2021; Muller et al., 2021; Suárez et al., 2021), various health problems (Ting et al., 2016; Welch et al., 2019; Dimitrijević et al., 2020), highlighting in the last 2 years, the COVID-19 pandemic (Eneyo et al., 2021; Mazierska and Rigg, 2021; Muller et al., 2021), social analysis (Carlini and Sánchez, 2018; Garcia, 2018; Allmark and Stratton, 2019; Fengjiang, 2021; Moraes and Ferreira, 2021), among others.

Studies that focus on nightlife, nightclubs, and their management are not very extensive, although the economic importance of nightlife is evident (Eldridge, 2019; Eldridge and Smith, 2019; Nofre, 2020). There are purely descriptive or historiographic studies of nightlife and nightclubs in Los Angeles (Hong and Duff, 1997), Belgrade (Todorovic and Bakir, 2005), Ios (Stylidis et al., 2008), Milwaukee (Campo and Ryan, 2008), Newcastle-Australia (Bavinton, 2010), London (Garcia, 2018), New York (Houser, 2018; Rees, 2019), Taiwan (Huang, 2011), Lisbon (Nofre et al., 2019), Sunny Beach (Tutenges, 2013), Ayia Napa (Sönmez et al., 2013), Corfu (Kamenidou et al., 2013), Perth (Allmark and Stratton, 2019), Wrocław (Iwanicki and Dłuewska, 2015), Berlin (Garcia, 2018), Barcelona (Nofre et al., 2018), Madrid (Aramayona and García, 2019) and Sydney (Homan, 2019), but without delving into systematic classifications. In the management-related field, there is a group of studies (Skinner et al., 2005, 2008; Kubacki et al., 2007) on servicescape (Bitner, 1992), some studies on the image of tourist destinations (Stylidis et al., 2008; Berrozpe et al., 2017) or premises (Rivera, 2010; Aagerup, 2020), studies on the background of the tourist experience (Taheri et al., 2016; Nghiêm-Phú, 2020), and studies on residents’ attitudes (Serra and Ramón, 2017; Ramón et al., 2021). These works are the main publications of interest for management and marketing academics, and no typologies have been found from a marketing and service design point of view.

It should be noted that casinos and gambling have much more academic literature than nightclubs and nightlife (Choi et al., 2019; Philander, 2019; Yang et al., 2020) despite the similarities between both types of offer, especially in terms of the negative social impacts attributable to both activities.

## Nightlife in Ibiza

The beginnings of tourism in Ibiza date back to the first third of the 20th century, but it was between the second half of the 1950s and 1970s when the greatest tourist development took place. The rapid growth of the sector was due to the fact that it made it possible to leave behind the previous situation of poverty, generating endogenous development with local entrepreneurs. The poverty experienced in the 1940s pushed many local people to start businesses linked to the new sector in order to improve their economic situation. To finance their initiatives, they resorted to savings or family properties and to request financing from tour operators or banks. They were entrepreneurs who made up for their lack of specialized training with enthusiasm and a great desire to learn. But despite their good intentions, mistakes were made that became visible in the 1980s. Tourism continued to grow rapidly until the growth of tourist sites came to a halt in the early 1990s and tourism volumes were stabilized by the year 2000, with tourism data more or less stable since then (Ramón and Serra, 2014; Ramón and Ramón, 2021).

From its beginnings, Ibiza grew protected by an image of freedom that attracted the European artistic avant-gardes at first and, later, beatniks and hippies (Ramón and Sánchez, 2022). In the 1950s, this atmosphere of freedom favored the existence of unthinkable parties, and not only in Spain. The first precursor bars of the future Ibiza nightlife, located in the port of Ibiza and in Sant Antoni are from that time. In the 1960s and 1970s, the festivals promoted by the hippie community took on an important presence and some of them have become a tradition that still continues. Between the late 1960s and early 1970s, the first nightclubs on the island opened (Ramón and Sánchez, 2022). The most significant one from that period was Pacha, located in the meadow on which the Promenade is located today, and Amnesia, on the road from Ibiza to Sant Antoni. In general, the nightclubs of the 1970s were small premises with a few workers and technical equipment that was adequate for the time, but modest. These venues grew rapidly in size, technology, and prestige, some of them reaching the present day (Ramón et al., 2015). Thanks to continuous reinvestments and improvements, some of the island’s nightclubs have exceeded 50 years of history, something very difficult in this sector.

The image of Ibiza would be difficult to explain without the boom of nightclubs in the 1980s. There was an increase in the number and size, driven by the expansion of nightclubs in the United Kingdom. In the 1980s, Pacha, Angel’s, Playboy, Amnesia, and Ku were internationally famous tourist attractions. Ku (today Privilege) in Sant Rafel stood out among these nightclubs, as a reference point and symbol of the island’s nightlife (Ramón and Sánchez, 2022). Ku was large in size (Privilege subsequently entered Guinness World Records for it), and in the originality and spectacularity of its events. At the beginning of the 1990s, there was a decline in this boom, partly due to the changing trends in the United Kingdom and to the tourism crisis and new regulations requiring nightclubs to be covered to reduce environmental noise. In the 1990s, an attempt was made to correct the image of a tourist destination of debauchery and partying so as not to scare off other types of tourism. Despite this, in the 1990s, a group of seven clubs was formed, which were the image of Ibiza (Amnesia, Eden, Es Paradís, Pacha, El Divino, Privilege, and Space). Since the end of the 1990s, restrictions have been applied to nightclubs to avoid the inconvenience they cause to residents and new formulas for nightlife premises have become popular, such as beach clubs and hotel clubs, which avoid some of the restrictions applied to nightclubs (Ramón et al., 2015; Ramón, 2019).

Throughout the first two decades of the 21st century, some premises have closed down and others have opened. Many of the new openings are completely new concepts or variations on previous ones. Hotel clubs appeared in 2008 with Ibiza Rocks, but became hugely popular in 2011 with the opening of Ushuaïa (Ramón, 2016; Ramón and Sánchez, 2016). Beach clubs were a scarce offer, led by Bora Bora in Platja d’en Bossa and Café del Mar in Sant Antoni, but with the new century, they have increased in number and sophistication (Blue Marlin, O Beach, etc.). Currently, the traditional bars and restaurants located on the beach have disappeared on some small beaches, being replaced by beach clubs. El Divino club opened in the 1990s and was successful for two decades, but due to the presence of the nearby Pacha club, it closed and the place was bought by the Pacha Group, which reopened as Restaurant Cabaret Lío. There has also been an increase in the presence of pleasure boats that hold parties on board with electronic music and alcoholic beverages (party boats). In fact, in recent years, the incorporation of musical events has become a common strategy in many venues (Ramón et al., 2020).

**Figure 1:**
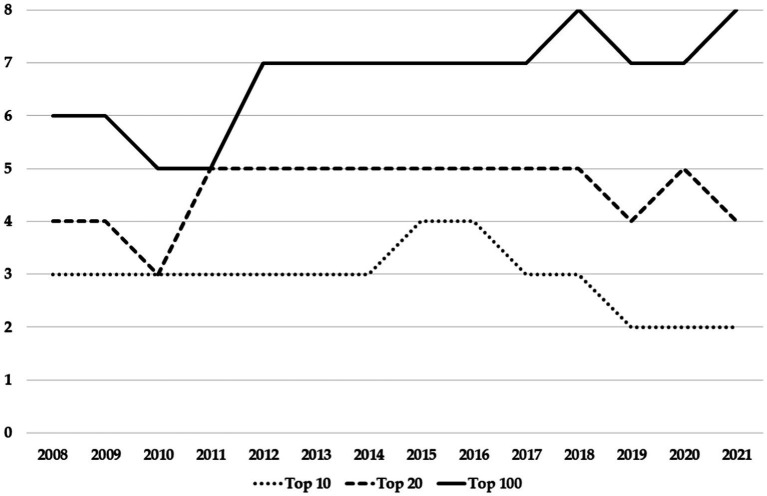
Ibiza Clubs in the Top 100. Source: DJMag (2022) and own elaboration.

The relevance of the nightclubs in Ibiza can be seen in several references in the sector. Within the International Dance Music Awards, the Best Global Club category was created in 2005 (Winter Music Conference, 2022). In the 15 editions of the Best Global Club category, Ibiza clubs have won 13 times: Space (2005, 2006, 2012, 2013, and 2014), Amnesia (2007, 2008, 2009, and 2011), and Ushuaïa (2015, 2016, 2018, and 2020). In the remaining two cases, they were clubs of high international prestige: Ministry of Sound London (2010); Space Miami (2019). DJMag magazine, a benchmark within the sector, has been publishing a ranking of the best 100 clubs in the world since 2004 (DJMag, 2022). Since the beginning, this ranking has included Ibiza’s clubs (Figure 1). The 2021 ranking follows the same trend with two clubs in the Top 10, four in the Top 20 and eight in the Top 100: Hï and Ushuaïa in the Top 10; Amnesia and Pacha in the next ten positions; and another four clubs (DC-10, Privilege, Eden, and Octan).

Despite the importance of Ibiza’s nightclubs, existing academic studies have the same approach as in the rest of the world, being a context for studies on alcohol and drug consumption (Bellis et al., 2009; Hughes et al., 2009; Kelly et al., 2014), violent behavior (Hughes et al., 2008, 2009; Calafat et al., 2013) and risky sexual relationships (Hughes et al., 2009; Downing et al., 2011; Kelly et al., 2014). These studies prioritize the analysis of British tourism, especially in Sant Antoni, from the point of view of British researchers, although in recent years comparisons have been made between tourist-sending countries (Hughes et al., 2008; Downing et al., 2011).

Surprisingly, the local vision of this activity and analyses from areas, such as geography and economy, are still rather absent, although there are already some exceptions (Serra and Ramón, 2017; Capellà, 2018, 2020; Ramón et al., 2020). In any case, there are still many topics to be studied regarding nightlife, both in Ibiza and the rest of the world.

## Methodology

The methodology used is based on the case study (Yin, 2017) and the classification of the premises has been made taking into account: the legal form in which they are registered (nightclub, bar, restaurant, hotel, yacht charter, etc.); the current or potential international relevance of the establishment based on the DJMag list (Top 100 Clubs; DIMag, 2022) and the International Dance Music Awards (IDMA) of the Winter Music Conference (Best Global Club category; Winter Music Conference, 2022); the differences in the offer (much higher prices, sunset as an attraction, relevance of parties held, etc.) and target market (tourists with high purchasing power, homosexuals, middle-aged people, etc.). In the classification, the characteristics of the commercialized product have prevailed over the legal form.

The diversity of the offer detected has made it necessary to create a definition that allows the object of study to be delimited. This paper analyses premises in Ibiza that offer musical events on a regular and planned basis, and these events are of great importance for promotion. These scheduled events consist of live music, with styles ranging from jazz to rock, to chill-out, classical or electronic music. Disc jockey (DJ) events would also fit the definition, provided there is onsite artistic mixing. Venues with occasional events or that offer music on a continuous but prerecorded basis, for example musical threads or playlists, are excluded. All this implies permanently having a suitable sound system and a mixing console. Although traditionally referred to as “nightlife,” the set of types analyzed can cover 24 h a day, with both night and daytime offer. In summary, the main feature is the regular offer of live music used as a differential element.

Nightclubs have parties and musical events as their main offer, while the other formats have another activity as the main one, with events being a differentiating element. The regular presence of an establishment among IDMA candidates (Winter Music Conference, 2022), or among the top 20 in the DJMag ranking is considered of maximum relevance and sporadic presence in both lists is considered of moderate relevance. Prices are considered very high when they are double the prices of equivalent services in similar premises. The information for this classification has been obtained from the local press, bibliography, and company websites or websites specialized in leisure, in addition to visiting premises and tourist areas. This has allowed for the creation of a profile of the different types of premises in the sector, indicating the most significant elements of each type of offer.

## Classification of the Nightlife Offer in Ibiza

Innovation has enabled the island’s leadership in a very complex sector and has led many new nightlife offers to have their origins or some of their first examples in Ibiza. For example, music bars located in the port were among the most innovative and advanced in the 1960s. In the 1980s, the island’s big nightclubs were a European and world reference. Since the 1990s, the most important nightclub parties have their own brand image and are a product in themselves, which is taken to nightclubs all over the world and have their own merchandising lines. A beach club like Café del Mar was an absurd idea until its launch proved to be a success. Hotel clubs emerged in the new century, which were considered a very risky idea at the beginning, and today are seen as a success to imitate. This and more has resulted that over the last 40 years, the Ibiza season has become the benchmark for nightlife in the rest of the world all year round. All this implies that Ibiza’s premises have great relevance and prestige both among professionals in the sector and electronic music fans, creating a trend in the sector.

**Figure 2:**
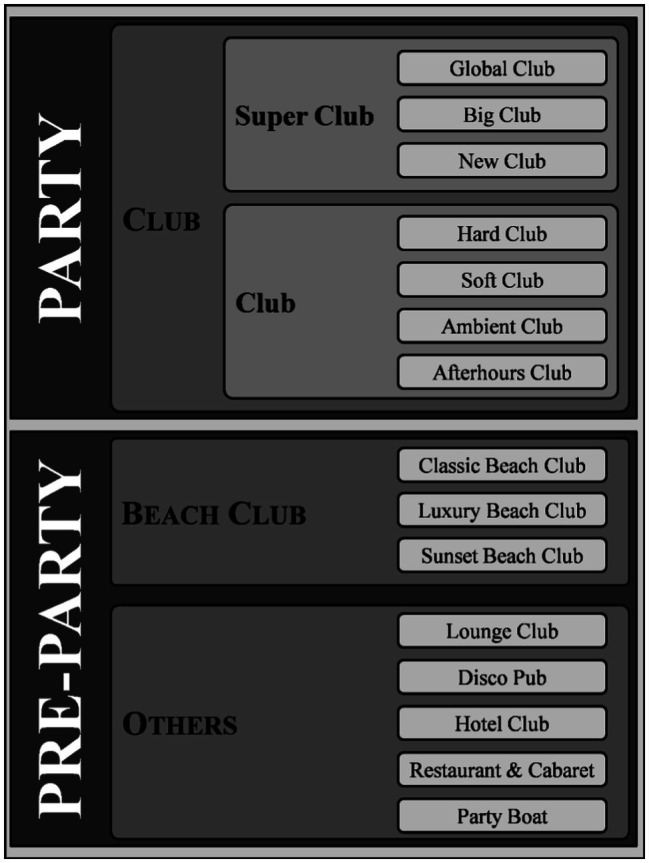
Classification proposal. Source: own elaboration.

When going into the details of Ibiza’s leisure offer, the first thing that can be said is that it can be classified according to its characteristics at different levels of disaggregation (Figure 2). In general, nightlife premises can be divided into two large groups according to the main hours of their activity: until midnight (Pre-Party), and after midnight (Party). It should also be noted that the first group are premises that do not have nightlife as their main activity, but as a differentiating element, and the second group has nightlife as its main activity.

### Main Nightlife Premises (Party)

These premises are characterized by having their peak after midnight and are the central element of nightlife. They are registered nightclubs as such and are subject to the corresponding regulations on capacity, security, and taxation. Due to the risk of accidents in crowded venues, they are the businesses with the best capacity control and evacuation plans. To this end, they have staff at the door who control access and act in the event of detecting violent behavior inside the club or at the doors. In terms of taxation, their main characteristic is that the normal Value Added Tax (VAT) is applied (currently 21% in Spain), while many businesses in the second group (Pre-Party) apply reduced VAT (10% in Spain). Until the emergence of the Ushuaïa club hotel in 2011, all the major venues in Ibiza with international projection belonged to this group.

#### Nightclub (Club)

**Figure 3:**
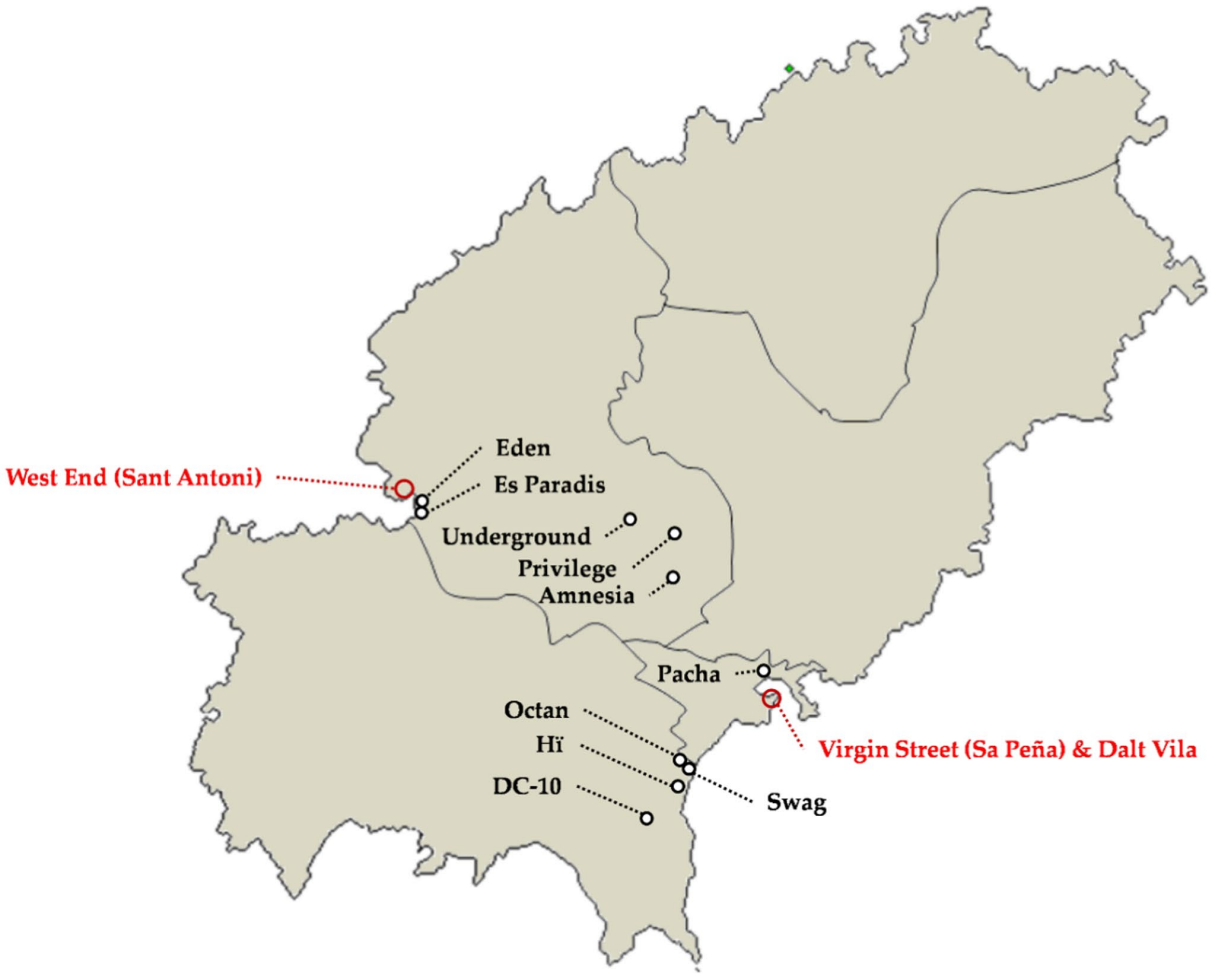
Macro-nightclubs and areas with Conventional nightclubs. Source: own elaboration.

The club or nightclub is the main element of the night offer (Figure 3) and can be of two types, depending on its international relevance. The first group is responsible for the international media impact, while the second group is a much more conventional offer and, although of good quality, it does not stand out from the usual clubs in other urban or coastal destinations around the world.

##### Macro-Nightclubs of International Prestige (Super Club)

These are large capacity venues in holiday destinations, such as Ibiza (1,500 to 10,000 people) and which have, or aspire to, an international presence. Internationally renowned nightclubs of urban destinations (Amsterdam, London, Berlin, New York, etc.) tend to be smaller in size (between 700 and 1,500 people). Macro-nightclubs structure their offer in a party calendar organized by international promoters as independent products. Parties, such as Cocoon, La Troya, Matinée, SuperMartXé, F*** Me I’m Famous, among others, have been held continuously for years and have a prestige independent of the club where they are held. These parties can change clubs from 1 year to the next without damaging their image, and they make annual tours in clubs around the world. They also have their own merchandising line and shops, such as SuperMartXé and F*** Me I’m Famous. In fact, nightclubs negotiate with DJs and producers to organize a season-long calendar of parties. The breakdown of this subtype can be increased by dividing the clubs into three subgroups.

##### Global Club

They are the largest and oldest (over 30 years of history in all cases), and are at the top of the world ranking: Amnesia, Pacha, Hï, and Privilege (Figure 3). All four clubs accumulate IDMA nominations and are among the top of the DJMag rankings on a regular basis. The capacity ranges between 3,000 and 10,000 people, being the largest clubs on the island. All of them have undergone a process of reinvestment, improvement, and expansion throughout their history. Amnesia appeared in 1975 and has changed ownership several times, but for several decades it has been in the hands of Martín Ferrer. Pacha Ibiza appeared in 1973 and was managed by Ricardo Urgell until its sale to an investment fund in 2017. Privilege opened in 1979 as Club San Rafael, but was soon renamed Ku and adopted its current name in the 1990s. Nowadays, Privilege is the largest nightclub on the island. Hï opened in 2017 under the management of Ushuaïa Entertainment S.L. (50% owned by Palladium Hotel Group), but that venue had previously been Space Ibiza, under the management of Josep Roselló (1989–2016). Therefore, Hï accumulates to its 3 years of history (in 2020 and 2021 the island’s nightclubs did not operate due to the pandemic) the 27 previous ones as Space club.

##### Big Club

They are big and veteran, but they only aspire to the Top 100 worldwide. Eden and Es Paradís (Figure 3) are in this group, which are both located in Sant Antoni. In both cases, their name refers to the biblical paradise from which humans were expelled. They have capacity for between 2,000 and 3,000 people, opened in the 1970s and are separated by a distance of barely 10 m. While Es Paradís has kept the same name and the same managers (currently in the hands of the second generation of the family), Eden has had different managers and names (formerly Star Club, Kaos, and Gatecrasher). They are important clubs, but they recognize that they cannot compete with the clubs in the previous subgroup.

##### New Club

They are newly created and aspire to Global Club or Big Club, occupying premises that have been used for this purpose on previous occasions. The main current example is Octan (formerly Sankeys), opened in July 2019 and with a capacity for 1,500 people (Figure 3). Ibiza is not a guarantee of success and the management of clubs is very complex because it depends on subjective perceptions and interactions between patrons. This has led to failures due to management and promotion problems, as is the case of Booom! (Formerly Angel’s, Penelope, Heaven, etc.). Booom! is the name under which a club located on the Promenade of Ibiza opened in its last attempt. Before that, it had already experienced different failures under different names and managers and, nowadays, the premises have already been demolished after several years without having any use. Swag (formerly Moma, Garbi, Martina, Essence, etc.), located in an annex of the Garbi Ibiza Hotel and Spa, also raises questions. In general, they are not very large venues (1,000–1,500 people) found in locations with major clubs nearby: Booom! was on the Promenade, very close to Pacha; Octan and Swag are in Platja d’en Bossa (Figure 3), very close to Ushuaïa and Hï. This problem was in fact the reason for the closure of El Divino (capacity for 700 people and located very close to Pacha). Finally, it should be noted that it is almost impossible to set up new large capacity clubs due to the opposition of social groups to this offer. All this, together with the enormous difficulty of managing these types of projects, make it unlikely that new clubs with the capacity to become a Global Club will appear.

##### Conventional Nightclubs (Club)

They are small nightclubs, of eminently local relevance, not very well known outside the island and do not aspire to international reference lists. In fact, their offer does not differ from that offered by clubs in population centers without the prestige of Ibiza and they do not have the capacity to attract tourists on their own, although they are frequented by tourists. They can be subdivided into several subtypes:

##### Hard Club

They are small with some experience in the sector and a reasonably good offer (live music or internationally renowned DJs), ranking immediately below macro-nightclubs. They offer parties that are potential alternatives to macro-nightclubs or, at least, a complement at the same level. A clear example is Underground located in Sant Rafel (Figure 3), near Amnesia and Privilege, but with a long history.

##### Soft Club

They are small with no outstanding elements and a low-level offer for a destination like Ibiza (hardly known DJs and rarely live music). They are the most basic premises in their offer and are frequented by tourists in summer and residents in winter (those that are open all year round). There are many examples, mainly in the West End of Sant Antoni (Figure 3): Casanova, Hogan’s, Hot, Koppas, La Noche, People, Simple, etc.

##### Ambient Club

In Calle de la Virgen in the Sa Peña neighborhood of Ibiza and in the Dalt Vila neighborhood in the LGBT area (Figure 3), highlighting several small long-established premises focused on this public (Angelo, Dôme, Lola’s, S’Amphora, etc.). They are very small, old, focused on a very specific market niche, and hardly known to the general public. It should be noted that the island’s parties are mostly considered gay friendly and this means that the specialized offer is scarce.

##### Afterhours Club

Opening and closing hours are fairly controlled, but the afterhours offer (parties held after the official closing time) has a long tradition on the island and special permits are still requested for opening and closing nightclub parties. The pioneers in this offer were Amnesia in 1985, Ku in 1986, Space in 1989, and DC-10 in 1999, combining both types of offers and staying open for up to 22 h a day. For many years, Space combined the regular and afterhours offer, although the Ibiza Island Council banned afterhours in 2008. However, the establishment most related to afterhours, since it opened in 1999, has been DC-10 (Figure 3). DC-10 started with a purely afterhours offer and for many years experienced frequent temporary closures for non-compliance with regulations. Currently, DC-10 is a nightclub with conventional hours and quite a lot of international recognition, despite having much less promotion than other clubs on the island and being smaller (capacity for 1,500 people). At present, it can be said that afterhours parties have disappeared as a specialization or as a complement to Ibiza’s nightclubs.

### Complementary Nightlife Premises (Pre-party)

**Figure 4:**
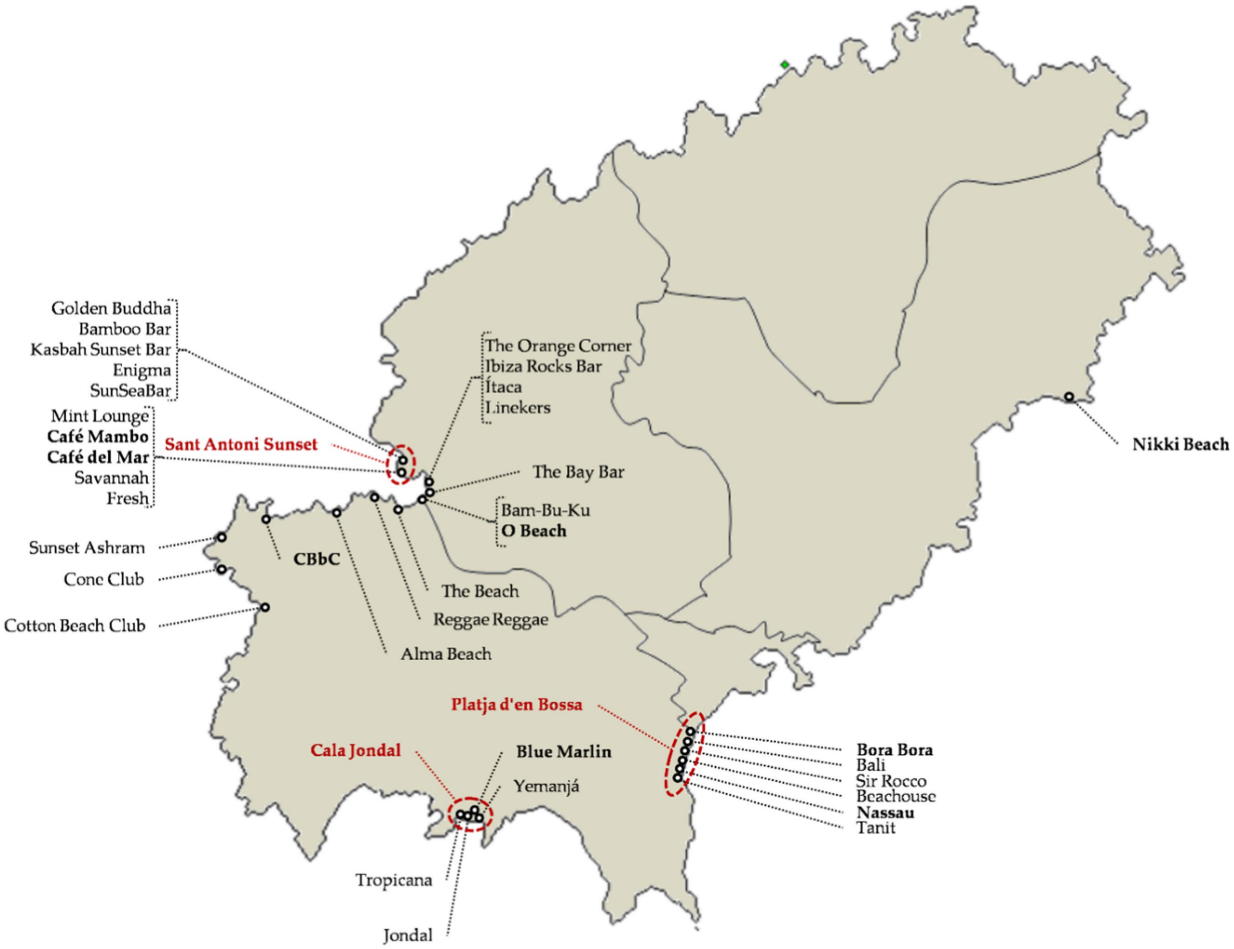
Main beach clubs. Source: own elaboration.

The previous group is made up of nightclubs that perform nightclub activity, with greater or lesser success. This group is made up of premises that are not nightclubs, but which have incorporated musical events as a differentiating element in their offer. These are innovations that assimilate bars, restaurants, hotels, and boats to nightclubs. In some cases, they are popularly called nightclubs, but they are somewhat different and operate in different time zones to nightclubs. The premises of this group operate mainly between mid-afternoon and midnight, preceding nightclub parties. Beach clubs stand out for their abundance (Figure 4) and importance and, therefore, they are treated separately from the rest of the offers, which are much smaller and recent.

The pioneering businesses of each type faced high levels of risk and uncertainty when proposing unprecedented projects and totally subject to the subjective perception of potential patrons. These venues have revived open-air parties, traditional in the nightclubs of the island until the 1990s, but this has two implications: noise regulations force them to end before midnight; there are collectives that are critical of these offers, considering them tools to evade the regulations that control nightclub activities. They have also been criticized by nightclubs for being subject to different rules that give them advantages.

#### Beach and Waterfront Premises (Beach Club)

Beach clubs are an evolution of the food and drink premises located on beaches (Figure 4), and offer music together with a much more sophisticated service and atmosphere than traditional beach premises. They also have higher prices than traditional premises. Although the first one on the island appeared in the 1980s, it is from the year 2000 onwards that they achieved great international relevance and increased in number, almost eradicating traditional premises on some beaches (for example, CBbC in Cala Bassa). Within Ibiza’s beach clubs there are three main approaches.

##### Classic Beach Club

It is the most traditional and basic approach, offering electronic music, food, and cocktails on the beach. The area with the most presence of this classic format is Platja d’en Bossa, in the southeast of the island (Figure 4). The best-known examples are Bora Bora and Nassau Beach Club (Figure 4). The most emblematic is Bora Bora (opened in 1986) and it is the result of trying to contribute “something more” to the bar service that was located on the ground floor of Ibiza Jet Apartments, in Platja d’en Bossa. The result is that Bora Bora is internationally known, but the tourist apartment building in which it is integrated is almost unknown. Other more recent beach clubs are Nassau, Tanit, Bali, Beachouse, and Sir Rocco (Figure 4). The latest appearances are the result of a change of ownership and management in previous beach clubs.

##### Luxury Beach Club

As for the classic approach, it involves more luxury and much higher prices. In fact, in recent years there has been an increase in prices and an imitation of styles that have made many of the new beach clubs be candidates for this category, for example Nikki Beach and CBbC (Figure 4). However, the most representative premises of this approach are Blue Marlin, located in Cala Jondal, and O Beach, which is in Sant Antoni Bay (Figure 4). Traditionally, access to beach clubs, as they are in fact bars, cafés, or restaurants, was free of charge and without the need for entrance or door staff, but in recent years, some of the most exclusive beach clubs have adopted restriction policies in their access.

##### Sunset Beach Club

Sunset Beach Club to the classic approach they add sunset contemplation as its main attraction. They are located in the western part of the island, mainly the Sant Antoni Sunset and Sant Antoni Bay (Figure 4), and although there are many premises known for this offer (Café Mambo, Golden Buddha, Savannah, The Orange Corner, etc.), the main one is Café del Mar (Figure 4), being the promoter and dean of this commercial offer that recalls hippy customs inspired by primitive solar cults. Café del Mar has its origins in a bar located on the ground floor of a small tourist apartment building in Sant Antoni. This bar was not even located next to a beach, but on a rocky area of the coast. The idea of using sunset contemplation as an attraction seemed crazy or somewhat absurd, but the success of Café del Mar for 40 years and the large number of “imitators” show that it was not.

#### Other Music and Nightlife Options (Others)

**Figure 5:**
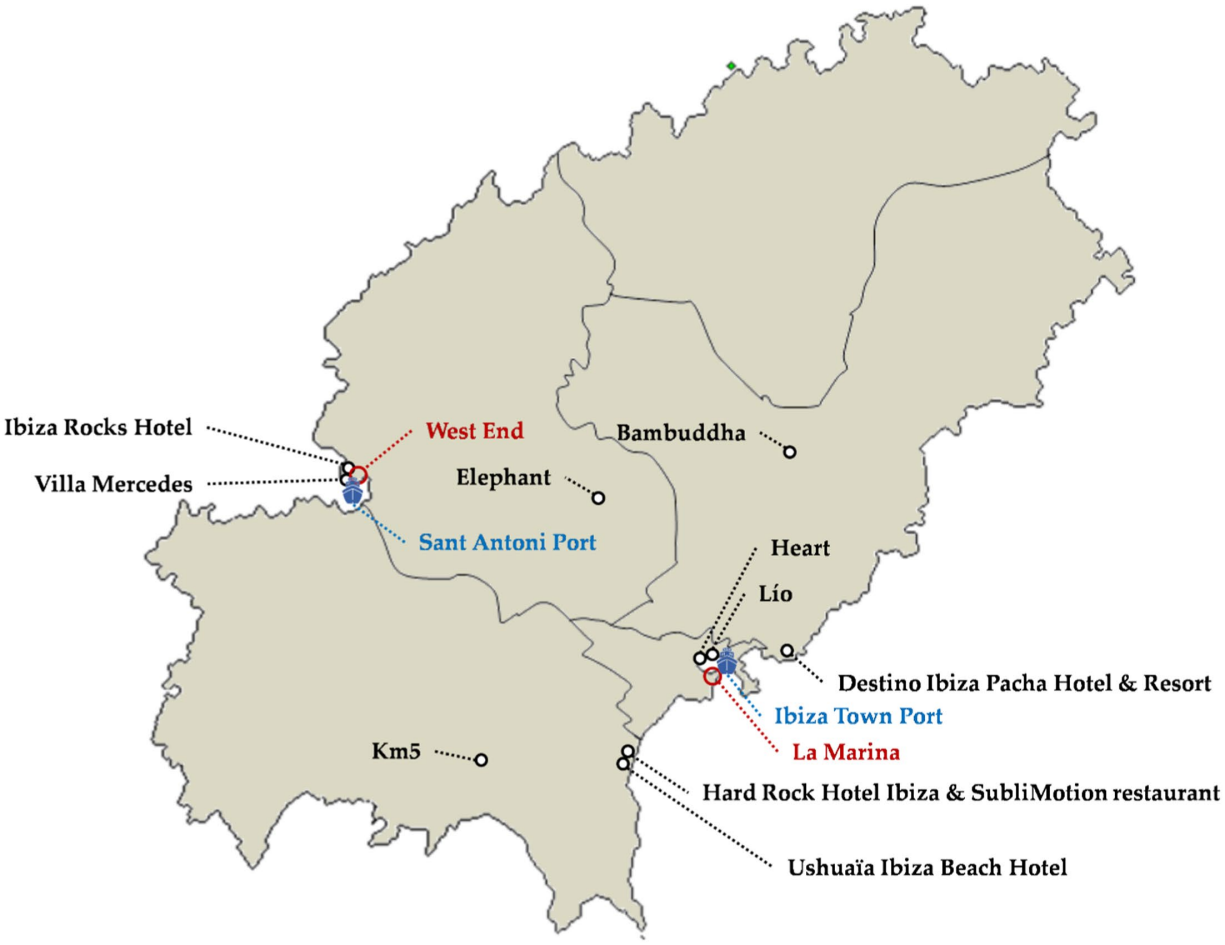
Other music and nightlife options. Source: own elaboration.

The range of nightclubs and beach clubs is extended by these other premises (Figure 5). They are less important offers from the media or quantitative point of view, but they complement the main premises of the leisure offer.

##### Lounge Club

Lounge Clubs are restaurants and bars with chill-out music and a more or less oriental inspired atmosphere, in a way heirs to the hippie spirit of the 1960s and 1970s. Examples of this offer are Bambuddha, Km5, Elephant, and Villa Mercedes (Figure 5), some of them with many years of history. Their clients are older than in other cases and seek to spend the night in a relaxed way, having dinner, drinking, and chatting to friends. In fact, it is an offer that lies on a blurred borderline between a conventional restaurant and a conventional nightlife establishment.

##### Disco Pub

Disco pubs are cocktail bars with electronic music and DJs. They are the result of the tendency to incorporate this type of music in all kinds of premises to liven up the night. The main examples are found in Sant Antoni (West End area) and Ibiza capital (La Marina and port; Figure 5). The festive atmosphere in both areas was boosted by the parades that promoted parties in macro-nightclubs, but in recent years these parades have disappeared due to municipal regulations. This type of offer is the oldest and has its origins in the 1950s and 1960s, when the Ibiza Port, a few bars in Sant Antoni, and private parties were the only nightlife offer on the island.

##### Hotel Club

Hotel clubs are hotels that have specialized in organizing large music events (electronic at Ushuaïa, rock at the Hard Rock Hotel, or British rock and indie at Ibiza Rocks) in their premises, generally in the pool area. When parties are held in the pool area, which is an open space, they must legally end before midnight or risk significant penalties. It is a recently created format, the first on the island being Ibiza Rocks in 2008 and Ushuaïa in 2011. Ibiza Rocks is a project by Andy McKay and Dawn Hindle based on the Manumission Rock party which they have organized since 2005 at Privilege or Bar M. Ushuaïa is a project by Abel Matutes Prats, in collaboration with Patrick Pissenem Yann, which took more than 3 years of preparation before its authorization because his father, Abel Matutes Juan, and the rest of the board of directors saw no future for the draft. There are currently four premises of this type: Ibiza Rocks Hotel, Ushuaïa Ibiza Beach Hotel, Hard Rock Hotel Ibiza, and Destino Ibiza Pacha Hotel and Resort (Figure 5). Ushuaïa is the best known and the most controversial, alleging that it is an open-air macro-nightclub (Ramón and Sánchez, 2016; Ramón, 2019). In recent years there have been no new openings for this type of offer.

##### Restaurant and Cabaret

Restaurants with entertainment have been opened recently, such as Lío, with a cabaret inspired show, SubliMotion (located in the Hard Rock Hotel Ibiza), which seeks to turn dinners into a show of perception and emotion, or Heart, created between the Adrià brothers and Cirque du Soleil (Figure 5). The Lío Restaurant Cabaret belongs to the Pacha Group and is the result of the conversion of the former El Divino nightclub. With Lío, the Pacha Group seeks to complement the offer of Pacha (nightclub), El Hotel (hotel boutique), and Destino (hotel club). The Heart restaurant is in the Ibiza Gran Hotel and offers a combination of haute cuisine and entertainment, but they have had to make adjustments to the business concept due to the poor results in its first years.

##### Party Boat

Party boats are boats that organize excursions of a few hours along the island’s coast and have incorporated DJs and a cocktail bar into the trip, being currently very controversial and subject to inspections due to the risks involved in holding parties on small boats at night and because of the inconvenience caused to the residents of coastal areas. Some of the parties held in party boats are linked to nightclubs or macro-nightclub parties, offering the party boat as a complement. Their main departure points are the ports of Sant Antoni and Ibiza town (Figure 5).

## Conclusion

Ibiza is a benchmark in the nightlife sector. Ibiza’s offer is not only made up of nightclubs, as many other types of premises have incorporated DJs, electronic music, and other elements typical of nightclubs. All of them have used music as an innovation to differentiate themselves from the competition. It can be said that in the last 10 or 15 years, innovation in leisure in Ibiza has consisted of putting on music and DJs. This new situation leads to proposing a first division between nightclubs (Party) and the rest of the offer (pre-party): bars, pubs, hotels, restaurants, boats, etc. However, even within clubs, there are very significant differences of approach (Hard, Soft, Ambient, After Hours, etc.), size (Club and Super Club), and prestige (Big Club, Global Club, etc.). This heterogeneity in offer makes it very difficult to quantify the sector, and the few published data are biased or inaccurate.

The main difference between the two large groups is that nightclubs are premises whose main activity is nightlife, charging entrance fees (VIP lists deserve a separate mention), and the second group (Beach Club, Disco Pub, Hotel Club and Party Boat) are businesses whose main activity is not nightlife but hospitality (cafés, restaurants, hotels, boat trips, etc.), and they have adopted elements of clubs as differentiation, charging admission fees or not, depending on the case. The differences between the two groups extend to taxation, as clubs have to apply a VAT rate of 21%, while the rest apply a tourist VAT rate of 10%, which is considered unfair competition for nightclub entrepreneurs. This accusation of unfair competition has made coordinated actions within the sector difficult.

All these types of premises are the result of the mistakes and success of a group of entrepreneurs. Nightlife is a service whose outcome depends on the interaction between patrons, so the co-creation of the experience is a core element of the product. In addition, the evaluation of patrons depends on their subjective perception of various elements, many of which are difficult to control. It is possibly the most difficult sector to manage within the tourist offer and, therefore, some entrepreneurs deserve a mention for their contributions and success. Ricardo Urgell Martí is the founder of Pacha Group and responsible for launching the Pacha Ibiza nightclub (with a hundred franchises), El Hotel, and Lío. His son Hugo Urgell started the Destino hotel. In 2017, Ricardo Urgell sold the Pacha Group to the investment fund Trilantic Capital Partners for € 290 million. Since then, Ricardo Urgell has been retired from the sector. Josep Roselló Prats opened several nightclubs and dance halls in Sant Antoni in the 1970s and 1980s, but his big project was the Space Ibiza nightclub. When the lease on the Space club ended in 2016, he retired from the sector. Both Ricardo Urgell and Josep Roselló have more than 50 years of experience in nightlife management, were specialists in the sector, and were significantly successful. Another businessman worthy of mention is Abel Matutes Prats of Ushuaïa and Hï, both in collaboration with Patrick Pissenem. In addition, Martín Ferrer Casals can be mentioned for the international relevance achieved by Amnesia under his management.

Nightlife generates significant controversies among the local population due to its potential negative social impacts. The sector generates economic impacts and has helped to differentiate and enhance the international image of the island, but it also generates noise, traffic problems, and other annoyances for residents living near the clubs. Furthermore, this sector is often linked to: alcohol and drug abuse; the presence of prostitution in the region; increased aggression and fighting; among others. This means that the opinion on this offer is divided between supporters and detractors (Serra and Ramón, 2017; Ramón et al., 2021). Looking to the future, it is necessary to work so that this activity can coexist with residents´ needs, but bearing in mind that freedom is an essential element for the success of a key sector for the image and tourism offer of Ibiza.

The international importance of several premises and the frequency with which innovative formulas appear in Ibiza suggest that this classification can be used as a reference for classifying premises in other world regions. This classification can be repeated in other areas, both urban (Amsterdam, Bangkok, Barcelona, Beijing, Berlin, Las Vegas, London, Los Angeles, New York, Paris, Tokyo, etc.) and coastal (Bali, Camboriú, Cancún, Miami, Mykonos, Pag, etc.), both small and large. Although possibly, in other destinations, there are no examples of any of the categories: in inland destinations there will be no Beach Clubs or Party Boats; In areas with strict noise regulations, there will be little Pre-Party offer and in areas with tight schedule controls, there will be no Afterhours; in some regions, the LGBT offer is not possible for various reasons; in destinations with less international relevance, there will be no Global Clubs, Big Clubs or Super Clubs in general; etc. The limitations of this classification are essentially twofold: a marketing approach has been adopted to focus on the patrons’ vision; many types of offers are less than 10 years old and new types may continue to emerge, especially in the Pre-Party category.

Given that the existing literature on the management of the nightlife offer is still scarce, future lines of research will consist of studying further, through different methodologies (descriptive, causal analysis, etc.), the knowledge of the supply and demand of Ibiza and others similar tourist destinations (Bali, Mykonos, Pag, among others).

## Data Availability Statement

The original contributions presented in the study are included in the article/supplementary material, further inquiries can be directed to the corresponding author.

## Ethics Statement

Ethical review and approval were not required for the study on human participants in accordance with the local legislation and institutional requirements. Written informed consent from the participants was not required to participate in this study in accordance with the national legislation and the institutional requirements.

## Author Contributions

JR-C, MS-F, AD-S, and JA-G: conceptualization, investigation, writing—original draft, preparation, and writing—review and editing. All authors have read and agreed to the published version of the manuscript.

## Funding

This publication has been funded by the Consejería de Economía, Ciencia y Agenda Digital de la Junta de Extremadura and by the European Regional Development Fund of the European Union through the reference grant GR21161.

## Conflict of Interest

The authors declare that the research was conducted in the absence of any commercial or financial relationships that could be construed as a potential conflict of interest.

## Publisher’s Note

All claims expressed in this article are solely those of the authors and do not necessarily represent those of their affiliated organizations, or those of the publisher, the editors and the reviewers. Any product that may be evaluated in this article, or claim that may be made by its manufacturer, is not guaranteed or endorsed by the publisher.
